# A novel parallel overlapping mode for complete ablation of large benign thyroid nodules in a single-session radiofrequency ablation

**DOI:** 10.3389/fendo.2022.915303

**Published:** 2022-07-26

**Authors:** Tao Wu, Bowen Zheng, Lei Tan, Tinghui Yin, Yufan Lian, Shicheng Xu, Jin Ye, Jie Ren

**Affiliations:** ^1^ Department of Ultrasound, Third Affiliated Hospital of Sun Yat-Sen University, Guangzhou, China; ^2^ Department of Otolaryngology-Head and Neck Surgery, Third Affiliated Hospital of Sun Yat−Sen University, Guangzhou, China

**Keywords:** benign thyroid nodules, complete ablation, radiofrequency ablation, overlapping mode, single-session

## Abstract

**Background:**

Radiofrequency ablation (RFA) has been widely applied in patients with benign thyroid nodules (BTNs), and complete ablation in a single-session treatment brings great benefits to patients. While how the ablation should be planned and performed to achieve complete ablation in a single-session treatment in large BTNs remains unknown.

**Purpose:**

To determine a more suitable ablation strategy for sufficient treatment in a single-session treatment.

**Materials and Methods:**

This retrospective study included 108 BTNs receiving RFA treatment. These patients were divided into two groups: group A using one insertion point with a fan-shaped overlapping mode and group B using multiple insertion points with a novel parallel overlapping mode. All the treatments used a hydrodissection approach and moving-shot technique. Contrast-enhanced ultrasonography (CEUS) was used to guide the supplementary ablation. Follow-ups were performed at 1, 3, 6 and 12 months. The rates of supplementary ablation, initial ablation ratio (IAR), the rates of complete ablation (CAR), treatment effects and complications between the two groups were compared.

**Results:**

The group B had larger treated nodules (10.2ml vs 6.4ml, *P*<0.001) than group A, while group B had a lower rate of supplementary ablation (21.6% vs 75.4%, *P*<0.001), especially in the BTNs with craniocaudal diameters ≥30mm (22.0% vs 100%, *P*<0.001). With the assistance of supplementary ablation, both groups achieved similar IAR (100% vs 100%, *P*=0.372) and CAR (94.7% vs 94.1%, *P*=1.000). Two groups showed similar VRRs at 12-month follow-up (77.9% vs 77.5%, *P*=0.894) and similar rates of complications (3.5% vs 2.0%, *P*=1.000).

**Conclusions:**

Needle placement using the multiple insertion points with a novel parallel overlapping mode would be easier to achieve complete ablation with less supplementary ablation, especially in large nodules.

## Introduction

Thermal ablation (TA) has become a widely used minimally invasive approach for the management of benign thyroid nodules (BTNs), including radiofrequency ablation (RFA), microwave ablation (MWA), laser ablation (LA) and high intensity focused ultrasound (HIFU) (1–3). RFA has been suggested as an alternative to surgery in some authoritative guidelines (3–6). Different from the previous focus on the volume reduction to relieve the nodule-related symptoms or cosmetic problems (7, 8), current researches of RFA on BTNs have more concerned about the control of nodule regrowth (9–11), since Sim et al. pointed out that increase of the un-ablated volume occurred in 57.4% of incompletely treated BTNs in 27.5 months, and 24.1% of these BTNs regrew in 39.9 months (9). Therefore, it’s important to ensure sufficient and complete ablation without impacting safety for BTNs in a single-session treatment to avoid additional medical costs and pain to patients.

Researchers have taken many efforts to achieve complete ablation in a single-session ablation on BTNs (12–14), such as hydrodissection technique, vascular ablation technique (15), contrast-enhanced ultrasonography (CEUS), moving shot technique (MST), and bipolar ablation with multiple overlapping shot technique (16). However, no study to date has been conducted to determine a basic issue that how the needle should be placed inside the nodule and how to plan the multiple overlapping ablation strategy for complete ablation. We believed that this basic issue would determine the adequate ablation, which has been proven in liver tumors that the appropriate needle placement was important, especially in large ones, to help achieve sufficient ablation effectively, especially for inexperienced ones (17–20).

Therefore, in this study, we first compared the complete ablation of different overlapping modes, in a single-session treatment, in different sizes of BTNs, with the aim to determine a more suitable ablation strategy for sufficient treatment, especially in large BTNs.

## Materials and methods

### Patients

**Figure 1:**
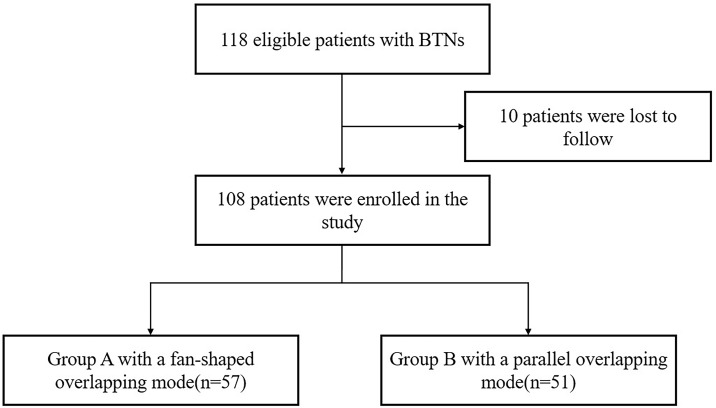
Patient enrollment process. BTN, benign thyroid nodule.

This retrospective study was approved by our ethics committee, and written informed consent was obtained from each patient. A total of 206 patients with BTNs underwent RFA in our institute from March 2016 to December 2019, and we included the patients with single or multiple BTNs that: 1) had one treated solid or predominantly solid nodule with the maximum diameter ≥20mm which was cytologically confirmed on US-guided fine-needle aspiration biopsy (FNAB); 2) had normal thyroid function; and 3) underwent one single-session RFA. The patients with substernal nodules were excluded. The selection of overlapping mode usually depended on the neck length, the nodule size and the patient wish, etc, and was finally determined by the operator when considering all these conditions. We informed the patient the selection before the procedure, and the possible additional needle puncture if supplementary ablation was needed for complete ablation. There were 108 patients eligible for the study, and these patients were divided into two groups according to the overlapping mode: group A with a fan-shaped overlapping mode and group B with a novel parallel overlapping mode (**Figure 1**).

### Operator and instrument

One clinician (J.R, with 6 years of experience in RFA) performed all the RFA procedures under the guidance of US using a GE LOGIQ E9 US scanner (GE Healthcare, Milwaukee, WI) with a ML2-9 linear transducer. We used a RFA device comprising VRS01 RFA system (STARmed, Korea) and internally cooled 18G electrodes with 7mm or 10mm active tips.

### Overlapping modes

**Figure 2:**
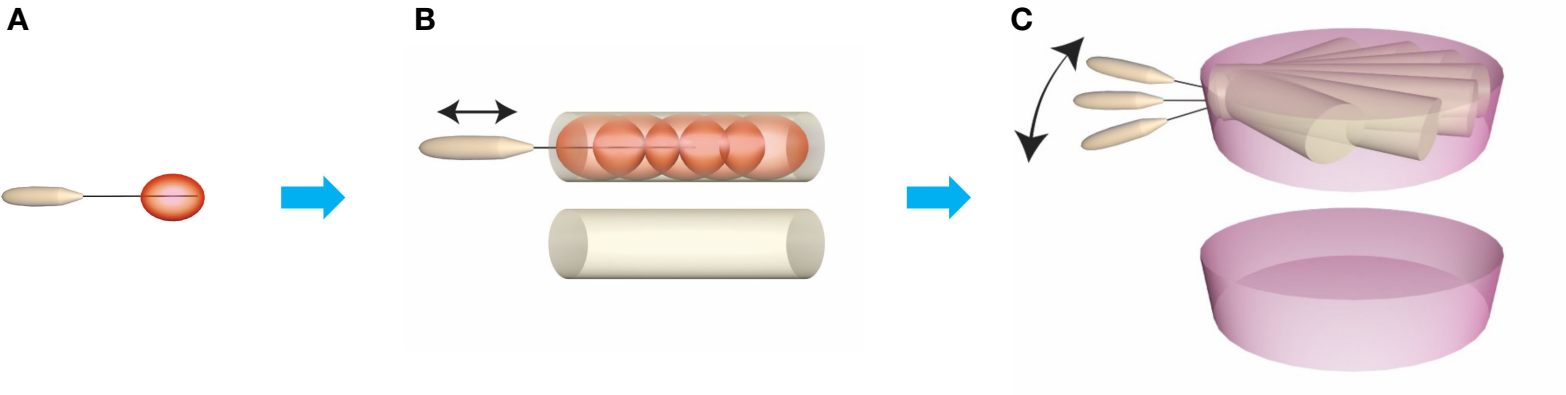
Construction of ablation plane unit. The basic ablation unit is generated keeping electrodes stable for 3-5 **(A)** and the ablation cylindrical unit is generated by moving the electrode on the same line from deepest part to superficial part **(B)**. Then, move the electrode on the horizontal axis making the ablation cylindrical units overlapping, and then the ablation plane unit on the horizontal axis is produced **(C)**.

**Figure 3:**
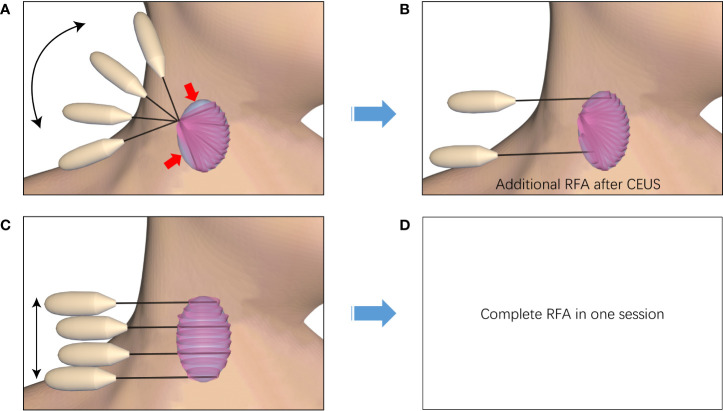
Diagram of needle placement strategies. The ablation planes are superimposed to achieve complete ablation of the entire nodule using one insertion point with a fan-shaped overlapping mode **(A)** or using multiple insertion points with a parallel overlapping mode **(C)**. The procedure using one insertion point with a fan-shaped overlapping mode may remain residual un-ablated areas which need to perform supplementary ablation **(B)**. The procedure using multiple insertion points with a novel parallel overlapping mode may achieve complete ablation **(D)**.

During the RFA procedure, local anesthesia, a hydrodissection approach (5% glucose), lateral approach, and an MST were used. US and contrast-enhanced ultrasonography (CEUS) were used to decide the ablation plan before the procedure. It was assumed that the ablated nodule could be virtually divided into multiple planes, and each plane into multiple lines, which were assembled by the multiple ablation units during the moving of the electrode inside the nodules. When the first plane was ablated, the needle was initially positioned in the deepest portion of the bottom line, and then moved backward slowly and continuously along the line (**Figure 2**). Once the first line had been treated, the needle was repositioned next to the prior line to create overlapping areas. It’s the same manner if the first plane had been done, the needle was repositioned next to the prior plane and created overlapping areas, until all the planes had been covered. According to the technique, using one insertion point on the skin should place the needle with a fan-shaped overlapping mode (group A, **Figure 3**) to cover all the planes of the whole nodule, and using multiple insertion points could place the needle with a novel parallel overlapping mode (group B, **Figure 3**). In the parallel overlapping mode using multiple insertion points, the distance between each point was calculated based on the thermal field of the radiofrequency ablation electrode. For example, the RF electrode with 10mm active tip could produce an ablation spheroid of 10mm*7mm*7mm when keeping in the place with power output of 45W for 3-5 seconds in solid nodules, so the craniocaudal distance between each insertion point was about 7mm. When considering the necessary overlapping, the distance between each insertion point was set approximately 5mm.

### CEUS-guided supplementary ablation and pain score during the procedure

When all the planned ablation planes had been done, CEUS was also used to determine whether the nodule achieved complete ablation, which was defined as complete non-enhancement in the whole nodule. If enhancement areas were shown in the nodule, which represented residual un-ablated areas, CEUS-guided supplementary ablation was performed until the whole nodule was treated or the safety margin was reached. Pain score during the procedure was recorded on a visual analog scale ranging from 0 to 10.

### Initial ablation ratio and complete ablation rate

IAR was determined by CEUS at 1 month after the RFA procedure, which was defined as the ratio of the ablated volume to the total volume of the treated nodule, using IAR (%) = ablated volume/total volume × 100% (21). The nodule was complete non-enhancement in the whole nodule on CEUS which indicates complete ablation. CAR (%) = the number of complete ablated nodules/total number of nodules × 100%.

### Follow-up

US and thyroid function test were performed at 1, 3, 6 and 12 months. The volume reduction ratio (VRR) of the nodules were calculated in US mode using: VRR (%) = (initial volume - final volume)/initial volume × 100%. Complications were recorded according to the definitions in the Society of Interventional Radiology (22, 23). We categorized symptom and cosmetic scores as defined in a previous guideline (5). A symptom score (SS) can be self-measured by patients using a visual analog scale (grade 0–10). Cosmetic scores (CS) were measured using the following scale: 1, no palpable mass; 2, a palpable mass with no cosmetic problem; 3, a cosmetic problem affecting swallowing only; and 4, clearly visible TN.

### Statistical analysis

Statistical analysis was performed with SPSS 20.0 package (IBM Corp., New York, USA). All reported P values are two-sided, and P values <0.05 were considered statistically significant. Quantitative variables are expressed as means ± standard deviations or medians (range). Differences between qualitative variables were assessed with a chi-square test or Fisher’s exact test. Differences between quantitative variables were analyzed with t test or Wilcoxon test.

## Results

### Group B had equivalent effectiveness but lower supplementary ablation rate than group A especially in large nodules with the craniocaudal diameters ≥30mm

**Figure 4:**
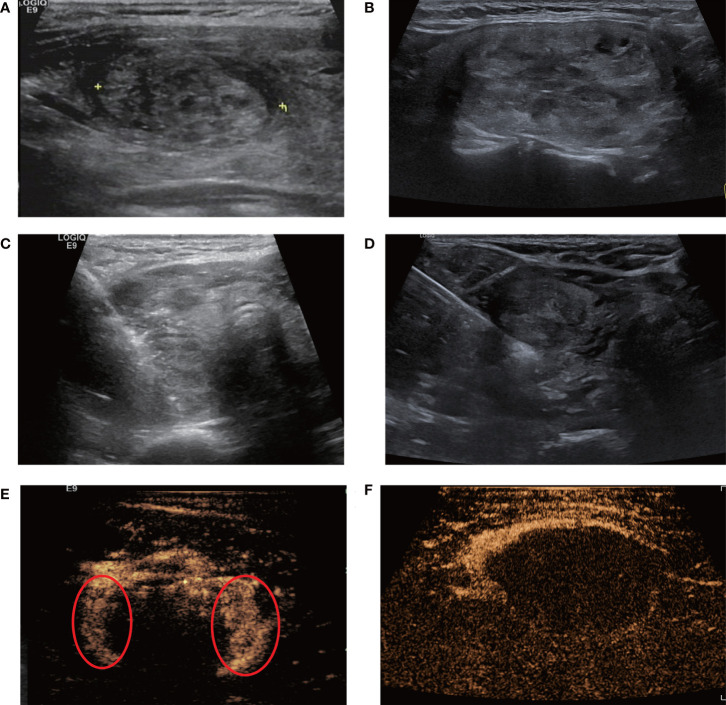
Single-session radiofrequency ablations of benign thyroid nodules using one insertion point with a fan-shaped overlapping mode **(A, C, E)** and multiple insertion points with a novel parallel overlapping mode **(B, D, F)**. **(A, B)** A 40-year-old female and A 46-year-old female A both had a predominantly solid nodule in the left lobe of the thyroid. Ultrasound examination showed the baseline volume of the nodule was 8ml, 14ml and the craniocaudal diameter was 3.0cm, 4.2cm, respectively. RFA was performed using one insertion point with a fan-shaped mode **(C)** or multiple insertion points with a novel parallel overlapping mode **(D)**. **(E)** Contrast-enhanced ultrasound after initial RFA using one insertion point with a fan-shaped mode: some tissue showed enhancement within the nodule, indicating residual tissue (circle). **(F)** The nodule using multiple insertion points with a novel parallel overlapping mode after initial RFA showed non-enhancement within the nodule, which meant that this mode could achieve complete ablation more easily.

**Figure 5:**
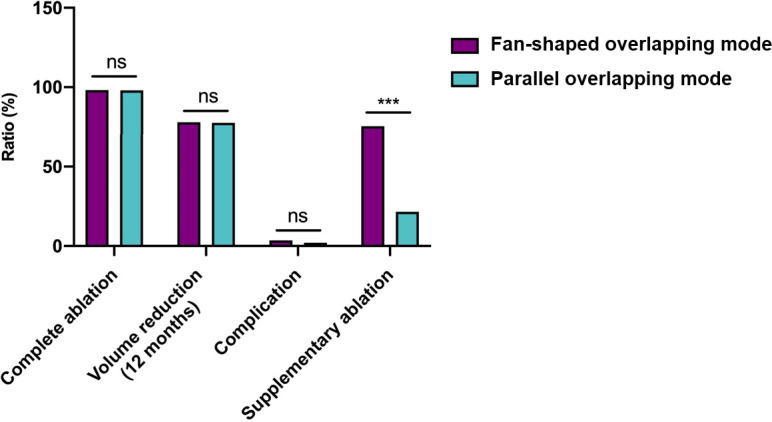
Graphs show the main treatment characteristics between needle placement strategies. ns, no significance. ***: *P*<0.001.

**Figure 6:**
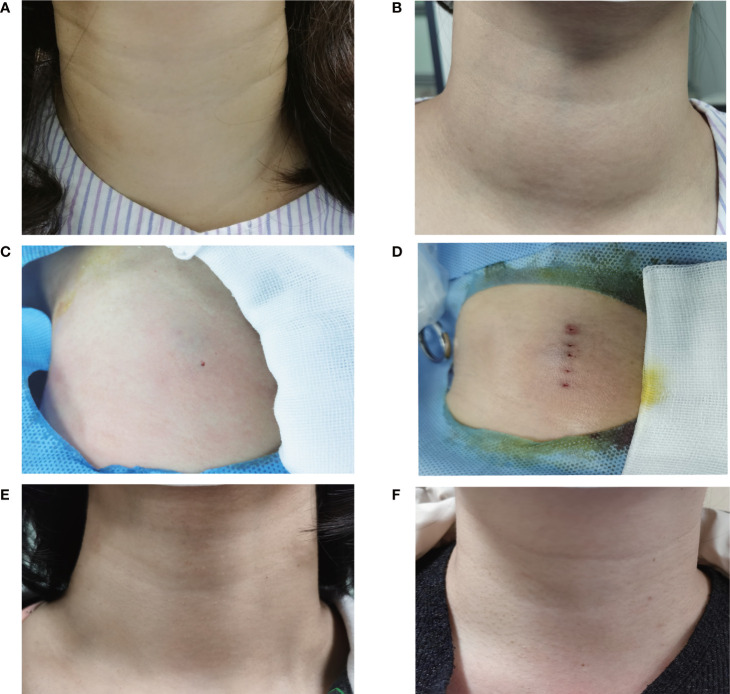
Comparison of the appearance of neck complaint using different needle placement strategies pre-procedure, on the procedure and at 12-month follow-up. The appearance of the fan-shaped overlapping and the parallel overlapping mode had a cosmetic score of 3 **(A)** and 4 **(B)** pre-procedure. The appearance of the fan-shaped overlapping mode and the parallel overlapping mode had one insertion point **(C)** and multiple insertion points **(D)** on the procedure. The symptom score and cosmetic score were significantly improved after RFA procedure. The patients both had a good cosmetic score without significant scars at 12 months after ablation using the fan-shaped overlapping mode **(E)** or the parallel overlapping mode **(F)**.

Among 108 patients with 108 BTNs, 57 patients (52.8%) were performed RFA using a fan-shaped overlapping mode (group A) and the remaining 51 (47.2%) using with a parallel overlapping mode (group B). The group B had larger treated nodules with larger maximum diameter (40.0mm vs 34.4mm, *P*=0.006) and larger volume (10.2ml vs 6.4 ml, *P*<0.001), while group A had more patients with residual un-ablated areas detected by CEUS and receiving supplementary ablation (75.4% vs 21.6%, *P*<0.001) to achieve complete ablation. Pain scores during the procedure were similar between two groups (3.2 vs 3.2, *P*=0.893). With the assistance of supplementary ablation, patients in both groups achieved similar IAR (100% vs 100% *P*=0.372) and CAR (94.7% vs 94.1%, *P*=1.000), and similar VRRs of 31.6% and 31.1% at 1-month, 53.0% and 61.0% at 3-months, 67.6% and 70.9% at 6-month, and 77.9% and 77.5% at 12-month follow-up, respectively (*P*=0.066-0.894) (**Figure 4**, **5**) (**Table 1**). The symptom score and cosmetic score were significantly improved after RFA procedure. The patients both had a good cosmetic score without significant scars at 12-month follow-up (**Figure 6**).

**Table 1:** Baseline characteristics and treatment characteristics of nodules of the patients and nodules in both groups.

	Group A (n=57)	Group B (n=51)	*P*
Age (years)	39.6 ± 11.8	43.3 ± 13.8	0.141
Sex (Female)	48 (84.2%)	45 (88.2%)	0.570
BMI	21.6 ± 2.8	22.8 ± 3.2	0.050
Maximum nodule diameter (mm)	34.4 ± 9.5	40.0 ± 11.6	0.006
Maximum nodule diameter ≥30mm	35 (61.4%)	41 (80.4%)	0.036
Nodule volume (ml)	6.43 (0.99-23.07)	10.22 (2.01-49.95)	<0.001
Nodule location (left/right/isthmus)	29 (50.9%)/27 (47.4%)/1 (1.7%)	25 (49.0%)/25 (49.0%)/1 (2.0%)	0.980
Nodule close to the dangerous structures			
Danger triangle area	42 (73.4%)	42 (82.4%)	0.279
Trachea	38 (66.7%)	41 (80.4%)	0.108
Esophagus	28 (49.1%)	24 (47.1%)	0.830
Carotid artery	40 (70.2%)	39 (76.5%)	0.461
Vagus nerve	15 (26.3%)	16 (31.4%)	0.562
FT3 (pmol/L)	4.21 (1.76-5.47)	4.41 (2.22-5.86)	0.505
FT4 (pmol/L)	13.09 (11.69-20.81)	13.25 (9.57-21.16)	0.329
TSH (mIU/mL)	1.48 (0.24-4.54)	0.98 (0.23-3.42)	0.058
CS	3.0 ± 1.1	3.3 ± 1.1	0.265
SS	2.1 ± 1.5	2.5 ± 1.6	0.163
Energy (KJ)	13.07 (4.06-56.65)	21.24 (4.19-96.20)	0.039
Ablation time (min)	50 (25–90)	45 (20-130)	0.926
Supplementary ablation	43 (75.4%)	11 (21.6%)	<0.001
Pain score during the procedure	3.2 ± 1.1	3.2 ± 1.4	0.893
IAR (%)	100 (85.7-100)	100 (80.5-100)	0.372
CAR(%)	94.7	94.1	1.000
1-month VRR (%)	31.6 ± 19.6	31.1 ± 19.1	0.893
3-month VRR (%)	53.0 ± 18.8	61.0 ± 17.9	0.066
6-month VRR (%)	67.6 ± 16.6	70.9 ± 15.0	0.456
12-month VRR (%)	77.9 ± 16.9	77.5 ± 15.6	0.894
12-month CS	1.3 ± 0.6	1.3 ± 0.6	0.782
12-month SS	0.2 ± 0.5	0.2 ± 0.5	0.805
Complications	2 (3.5%)	1 (2.0%)	1.000

Group A, patients with one insertion point; Group B, patients with multiple insertion points.

BMI, body mass index; FT3, free triiodothyronine; FT4, free thyroxin; TSH, thyroid stimulating hormone; CS, cosmetic scores; SS, symptom score; IAR, initial ablation ratio; CAR, complete ablation rate; VRR, volume reduction ratio.

When lateral approach was used, the needle would be adjusted in the craniocaudal axis. Therefore, sub-groups were categorized according to the nodule diameters in this axis. In this sub-analysis, group A had a higher rate of supplementary ablation in the craniocaudal diameters ≥30mm than that in <30mm (100% vs 36.4%, *P*<0.001), while group B did not show significant difference in both diameters (22.0% vs 20.0%, *P*=1.000). Besides, group B had a lower rate of supplementary ablation in the craniocaudal diameters ≥30mm (22.0% vs 100%, *P*<0.001) than that in group A, while both groups showed similar low supplementary rate (20.0% vs 36.4%, *P*=0.440) in the diameter of <30mm (**Table 2**).

**Table 2:** Sub-groups analysis of treatment characteristics in different maximum nodule diameters.

	<30mm			≥30mm		
	Group A (n=22)	Group B (n=10)	*P*	Group A (n=35)	Group B (n=41)	*P*
Nodule volume (ml)	3.46 (0.99-6.83)	3.23 (2.14-6.29)	0.878	11.06 (2.45-23.07)	13.16 (2.01-49.95)	0.026
Energy (KJ)	8.80 (4.06-30.92)	14.26 (4.82-23.42)	0.017	26.31 (7.75-56.65)	22.21 (4.19-96.20)	0.896
Ablation time (min)	41 (25-78)	38 (20-100)	0.678	60 (30-90)	49 (20-130)	0.085
Supplementary ablation	8 (36.4%)	2 (20.0%)	0.440	35 (100%)	9 (22.0%)	<0.001
IAR (%)	100 (85.7-100)	100 (100)	0.317	100 (98.0-100)	100(80.5-100)	0.152
1-month VRR (%)	29.4 ± 24.6	24.4 ± 21.0	0.592	33.0 ± 16.1	32.8 ± 18.4	0.959
Complication	0 (0)	0 (0)	1.000	2 (5.7%)	1 (2.4%)	0.592

Group A, patients with one insertion point; Group B, patients with multiple insertion points.

IAR, initial ablation ratio; VRR, volume reduction ratio.

### Two group both had a low rate of complications

There wasn’t any significant difference in the incidence of complications between both groups (*P*=1.000). Two patients (3.5%) in the group A experienced voice change and recovered within 1 month without any medication; and 1 case (2.0%) in the group B had local hematoma. All these three patients had nodules with the craniocaudal diameters ≥30mm. No patient had experienced infection, skin burn and nodule rupture. None of the patients experienced life-threatening or delayed complications during the follow-up period.

## Discussion

In this study, using the multiple insertion points with a novel parallel overlapping mode could achieve complete treatment with less supplementary ablation, especially in larger nodules with craniocaudal diameters ≥30mm. With the assistance of CEUS to detect residual un-ablated areas and guide the supplementary ablation timely, both groups could achieve similar complete ablation finally at the single-session treatment, and significant volume reduction at 12-month follow-up, with a low rate of complications.

Different from other solid tumors, BTNs have larger craniocaudal axis and adjacent critical structures. MST, which moves the needle tip inside the nodules during the ablation, is more suitable and commonly used for BTNs than fixed shot technique (FST) in other solid tumors (24, 25). Therefore, how the needle is placed and moves inside the nodules determines the accurate and conformal ablation, especially in the condition that a complete treatment should be achieved. A key step in needle placement is the insertion points on the skin. It’s easy to understand that when only one insertion point is used the needle should be placed as fan-shape during the shifts of the planes, while the needle could be placed parallelly if multiple points are inserted on the skin.

Though the previous researches rarely reported the number of insertion points (26), we could speculate that single point would be the most preference when considering that RFA is a minimally invasive treatment to leave as little damage as possible. However, the fan-shaped placement of the needle would be affected by the length of the neck and the position of mandible or shoulder. If a nodule with large craniocaudal diameter, or located near the upper or the lower of the lobe, the needle probably couldn’t reach the upper or lower margin because of the blocking of the shoulder or mandible to cover the whole nodule. Therefore, to achieve complete ablation by using this one insertion with fan-shape mode during a single-session treatment isn’t easy, and supplementary ablation should be needed. This could be proven in our study that this mode had a high rate of supplementary ablation (75.4%), especially in the larger nodules with craniocaudal diameters ≥30mm where the rate could reach 100%. Besides, this mode is highly dependent on the experience and stereoscopic knowledge of the clinicians, so that inexperienced ones should take time to receive special training. The RFA treatment in this study was performed by the radiologist with more than 5 years of RFA experience, which can ensure the smooth implementation of the needle placement strategy.

Multiple insertion points with a novel parallel overlapping mode could overcome the disadvantages of the one insertion mode mentioned above. BTNs could be easier to achieve complete treatment with less supplementary ablation by using this mode (21.6% vs 75.4%, P<0.001), especially in the larger nodules (22.0% vs 100%, P<0.001), since it’s seldomly affected by the size and location of nodule, the length or neck, or the position of mandible or shoulder. The spatial relation of the needle and the nodule could also be simplified under this mode, so that clinicians could plan the needle placement and perform the procedure more easily, achieve complete ablation faster, and may also relieve the initial nonconfidence.

Overlapping ablation is also an important reason for the needle placement mode and an important technique for complete treatment (16, 19). However, this technique was rarely mentioned in BTNs (16). The opinion that the basis of this technique is FST would be the possible reason. It can be seen that this technique largely depends on the careful planning and performing of the needle placement. Clinicians should take this into consideration when they decide the needle insertion and placement. Though multiple insertion points would inevitably cause a litter more damage on the skin or the subcutaneous tissue, the ablation of BTNs rarely causes needle tract seeding (23, 27, 28). Moreover, the rate of complications neither increased in our study (2.0% vs 3.5%, P=1.000), and the multiple insertion points gradually disappeared after follow-up, which did not increase the psychological pressure of patients. Therefore, for BTNs with larger craniocaudal diameters or in the sub-optimal location which could be affected by the neck structures, or for inexperienced clinicians, multiple insertion points with a novel parallel overlapping mode could be a better option.

Some limitations of this study should be considered. First, it’s a retrospective and non-randomized study. The preoperative selection of overlapping mode was finally chosen by the operator according to the neck length, the nodule size and the patient wishes, so that the selection bias couldn’t be avoided. Second, the lateral approach was chosen to access the nodule instead of the trans-isthmic approach, as for the nodule close to the recurrent laryngeal nerve, it is recommended through the lateral approach (29), which we considered easier to achieve complete ablation safely. Besides, we simply divided the cohort into two groups that single insertion point with a fan-shaped overlapping mode and multiple insertion points with a parallel overlapping mode, and other needle placements, such as multiple insertion points with a fan-shaped mode, weren’t discussed in our study. Further studies with prospective design, and more kinds of needle placement are needed.

In summary, two strategies of needle placement were first proposed in this study, and both could achieve similar complete ablation and treatment effect when combining overlapping mode and supplemental ablation. For clinicians, especially for inexperienced ones, it would be easier to perform the needle placement using the multiple insertion points with a novel parallel overlapping mode, and to achieve complete ablation with less supplementary ablation, especially in large nodules.

## Data availability statement

The original contributions presented in the study are included in the article/supplementary material. Further inquiries can be directed to the corresponding authors.

## Ethics statement

The studies involving human participants were reviewed and approved by Ethics committee of Third Affiliated Hospital of Sun Yat−Sen University. The patients/participants provided their written informed consent to participate in this study.

## Author contributions

TW, BZ, JY, and JR conceived and designed the study. LT, TY, YL and SX collected the data. TW, BZ, LT, and TY analyzed and interpreted the data. TW and BZ wrote the manuscript. All authors contributed to the article and approved the submitted version.

## Funding

This study is supported by the 5010 Clinical Research Project of Sun Yat-sen University (No. 2016016), National Natural Science Foundation of China (No. 81971632), Key Scientific and Technological Projects of Guangdong Province (No. 2019B020235002) and Natural Science Foundation of Guangdong Province (No. 2020A1515010425).

## Acknowledgments

We thank Zhicheng Yao (Department of General Surgery, Third Affiliated Hospital of Sun Yat-sen University, Guangzhou) for valuable discussion.

## Conflict of interest

The authors declare that the research was conducted in the absence of any commercial or financial relationships that could be construed as a potential conflict of interest.

## Publisher’s note

All claims expressed in this article are solely those of the authors and do not necessarily represent those of their affiliated organizations, or those of the publisher, the editors and the reviewers. Any product that may be evaluated in this article, or claim that may be made by its manufacturer, is not guaranteed or endorsed by the publisher.
